# Supplementary material for: Pygo-F773W Mutation Reveals Novel Functions beyond Wnt Signaling in Drosophila
Source: Int J Mol Sci. 2024 May 30;25(11):5998. doi: 10.3390/ijms25115998 (PMC11172468; doi:10.3390/ijms25115998)
Supplement: Supplementary file 1 [file ijms-25-05998-s001.zip › Supplementary Figure S1.pdf]

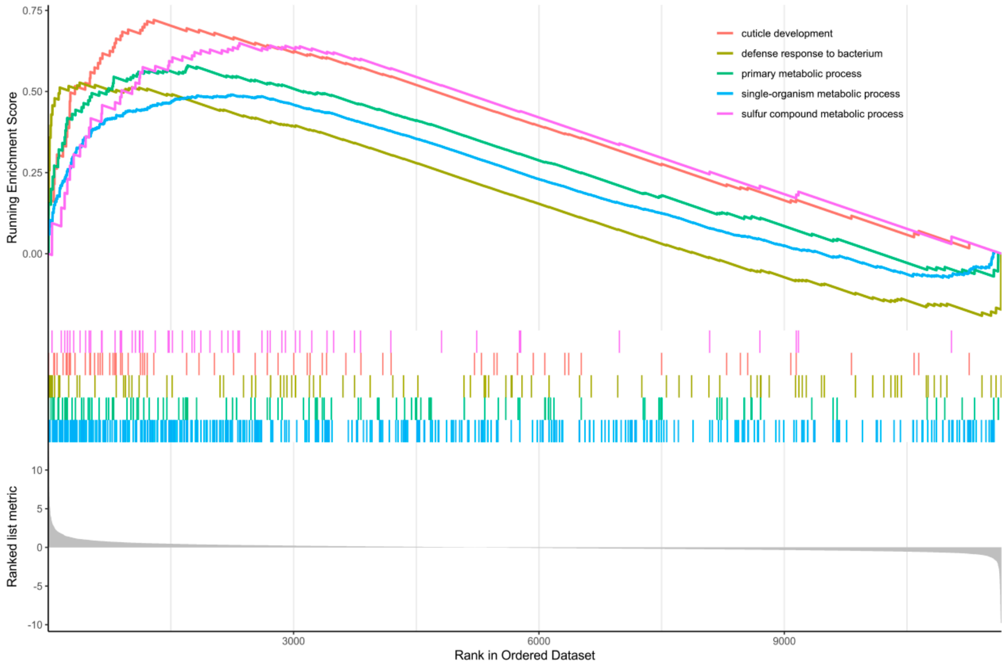

A: Biological_Process.top5. gseaplot

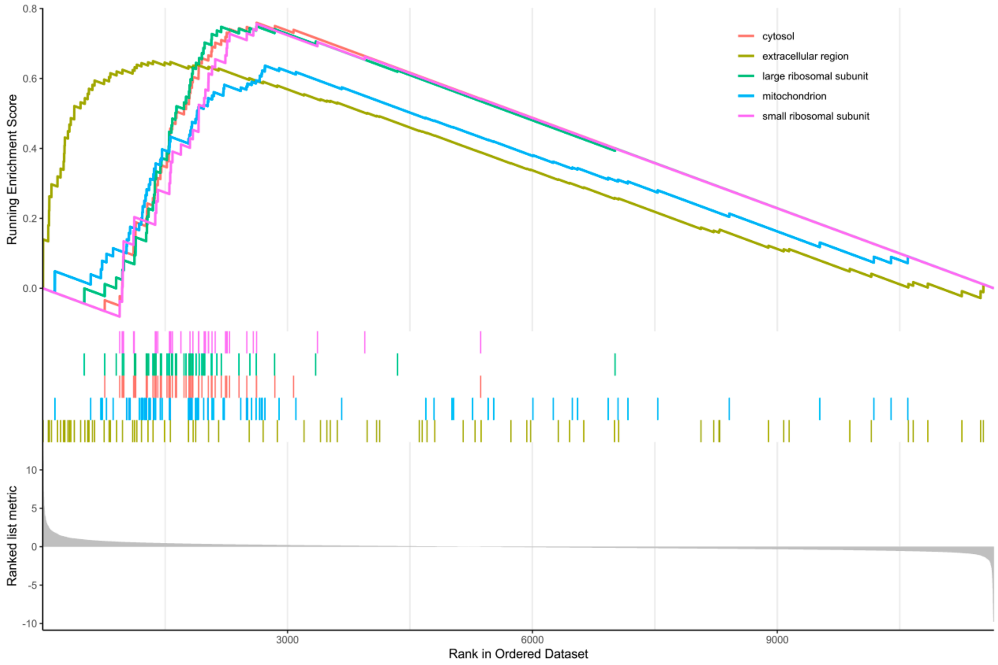

B: Cellular_Component.top5. gseaplot

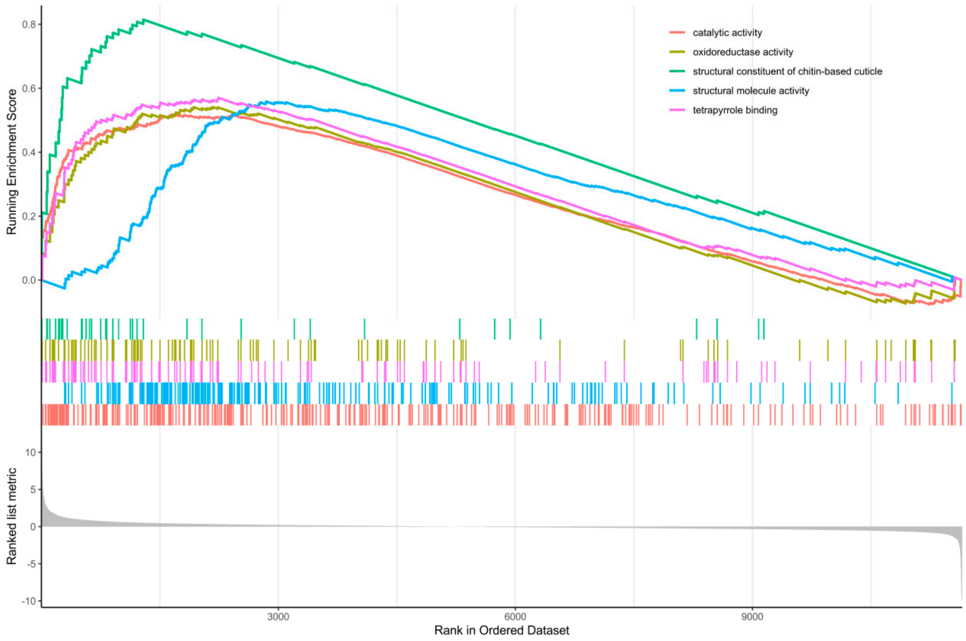

C:Molecular_Function.top5.gseaplot

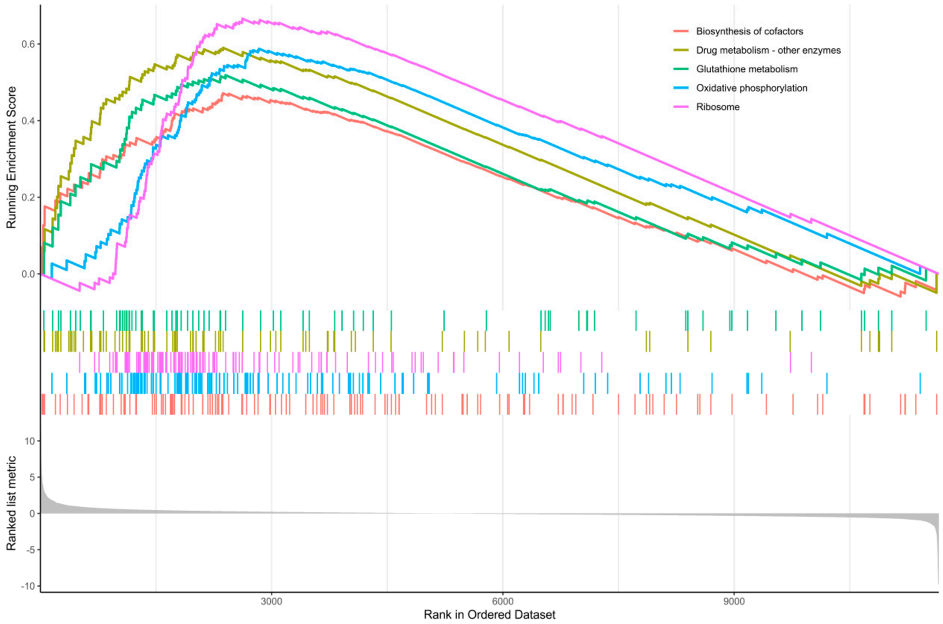

D: KEGG_pathway.top5.gseaplot

Figure S1 The top five pathways with the most reliable significance (i.e. the smallest pvalue) in Gene set enrichment analysis (GSEA) on Biological_Process, Cellular_Component, Molecular_Function, KEGG_pathway(A-D).
